# A novel progressive wave gyroscope based on acousto-optic effects

**DOI:** 10.1038/s41378-022-00429-4

**Published:** 2022-09-02

**Authors:** Lu Tian, Qiang Shen, Honglong Chang

**Affiliations:** Ministry of Education Key Laboratory of Micro and Nano Systems for Aerospacea, School of Mechanical Engineering, Northwestern Polytechnical University, Xi’an, 710072 China

**Keywords:** Sensors, Optical sensors

## Abstract

We propose and numerically investigate a brand-new, high-sensitivity progressive wave gyroscope based on acousto-optic effects for the measurement of rotational angular velocity. Unlike the traditional surface acoustic wave (SAW) gyroscope, which uses shifts in the SAW frequency to characterize the rotational angular velocity, this study uses acousto-optic effects to detect changes in refractive index caused by mechanical strain, measuring the angular velocity by the output optical power intensity of the optical waveguide. The three-dimensional finite element analysis method is utilized to build an SAW excitation model and optical detection model. We show that the sensitivity of the SAW gyroscope is highly dependent upon geometric parameters of the structure and that the mechanical strain induced by the progressive wave of the SAW can be effectively measured by the optical power intensity under the action of external angular velocity. The superiority of the proposed structure is substantiated by its achievement of a theoretical sensitivity of 1.8647 (mW/m^2^)/(rad/s) and high impact resistance of 220,000 g. By means of normalization, the sensitivity of the proposed structure can be enhanced by four orders of magnitude compared to the traditional SAW gyroscope. The novel structure combines the advantages of both conventional microscale vibrating gyroscopes and optical gyroscopes, providing a powerful solution for performance enhancement of SAW gyroscopes and, thereby, enabling application in the field of inertial devices.

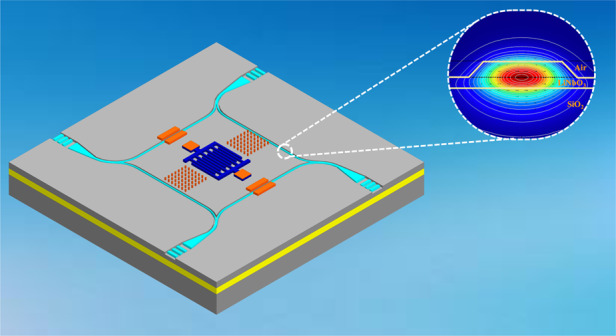

## Introduction

A gyroscope, as a device to detect external rotational angular velocity, can be combined with an accelerometer to constitute an inertial navigation system, which plays an irreplaceable role in many applications in military/industrial fields, e.g., inertial navigation, angular detection, and consumer electronics^1–5^. However, in some applications involving extreme environments, such as unmanned spaceflight, rocket launching, and oil drilling, the gyroscope’s chip is subjected to large impulsive loads^6,7^. For example, missiles undergo overloads of more than 20,000 g during launch, which cause the suspension-moveable elements of the gyroscopes to fail under the large load^8^. Detection structures such as sensing combs may be broken^9^, ingested, adhered^10^, or contaminated by particles^11^. It is difficult for the gyroscope to return to its pre-overload state; its performance is severely degraded, and its structure may even be comprehensively damaged, with the result that the gyroscope loses its working capability.

Through many years of development, scientists have proposed the utilization of surface acoustic wave (SAW) gyroscopes to solve the problem of overload. Completely different from microelectromechanical system (MEMS) gyroscopes, SAW gyroscopes usually adopt all-solid-state structures without any suspension-moveable elements^12^, which makes them able to withstand overload in extreme environments^13^. SAWs are a kind of wave that propagates along solid surfaces; they were discovered by British physicist Strutt in 1885 through seismic waves^14^. With the rapid development of SAW technology, SAW devices have been widely used in radar^15^, TV broadcasting^16^, wireless communication^17^, chemical sensing^18^, and other fields. Brand-new sensing mechanisms based on SAW technology have been a particular topic of intensive research. In 1977, Forst et al. first proposed the concept of the SAW angular velocity sensor. They used an external electromagnetic transducer to excite acoustic waves on the surface of the cylinder and detected the phase difference of SAWs during rotation to achieve the angular velocity measurement^19,20^. In 1980, Lao deduced the relationship between the wave velocity (*ν*) and the angular velocity (*ϖ*) on the rotating elastic medium, theoretically proved the gyroscope effect of SAW, and provided a new method for designing SAW angular velocity sensors^21,22^. Subsequently, Kueasava et al. proposed a novel SAW angular velocity sensor based on the standing wave mode, which generates Coriolis force perpendicular to the direction of velocity and rotation, obeying the Coriolis effect, and then excites the secondary SAW. The angular rate can be measured by this secondary SAW^23^. In 2000, Varanda et al. built the first prototype based on the standing wave mode and experimentally obtained a resolution of 0.038˚/s, which means that SAW devices have begun to move towards practical application of angular velocity measurement^24–26^. In contrast to the standing wave mode, Sang Woo Lee et al. demonstrated a progressive wave SAW angular velocity sensor that was developed based on the SAW gyroscope effect; they used a differential structure to achieve a signal output with a sensitivity of 0.431 Hz/(°/s)^27^. Wen et al. combined the characters of the standing wave and progressive wave and presented a new configuration of SAW angular velocity sensor based on the interference of intersecting SAWs, for a high sensitivity of 119 Hz/(°/s)^28,29^.

However, despite their great potential as a sensing mechanism, SAW gyroscopes still face many challenges, such as measurement limitation, sensitivity degradation, and temperature compensation. The standing wave SAW gyroscope outputs micro-/nanoscale electrical signals that are difficult to detect, and its orthogonal structures make it hard to compensate for temperature characteristics due to their substrate materials, thus ultimately affecting detection accuracy. In contrast, the differential structure of the progressive wave SAW gyroscope can realize temperature compensation, but the weak Coriolis force severely degrades the sensitivity. Although several methods have been developed to improve the sensitivity of SAW gyroscopes, such as high-order bulk wave diffraction built on a piezoelectric medium^30^ and phase detection based on SH wave modes^31^, the improved sensitivity still needs to be further enhanced to satisfy the practical application of angular velocity measurement. Fortunately, optical detection methods provide a new way of improving performance; their characteristics of high sensitivity, high stability, and low noise are helpful for accurate measurement^32–37^. According to the acousto-optic effect, optical detection methods and SAW technology can be combined to measure variances in the refractive index caused by mechanical strain in a solid medium, realizing gyroscopic out-of-plane angular velocity detection.

Based on this, we propose a new progressive wave gyroscope based on the acousto-optic effect. Through adopting single-electrode bidirectional inter-digitated transducers (IDTs) to excite the SAW on the surface of lithium niobate, analyzing the effect of structural parameters on gyroscope sensitivity, and utilizing acousto-optic diffraction to detect the mechanical strain induced by SAWs, the simulated results prove that the new method can significantly enhance SAW gyroscope sensitivity. In this paper, the principle and design of the progressive wave gyroscope, the acoustic process modeling, and the influence of the latter’s structural parameters on gyroscope performance are first introduced. In Section 3, a sensitivity expression of the gyroscope is obtained by analyzing the acoustic and photonic processes; the acousto-optic effect is analyzed by an finite element method model, and the impact resistance of the sensor is also analyzed. Through finite element simulation analysis, it is proved that the angle measurement of gyroscope can be realized by using the acousto-optic effect. In Section 4, this paper is summarized and compared with the performance of the existing literature.

## Results and discussion

### Device design and simulation

**Fig. 1:**
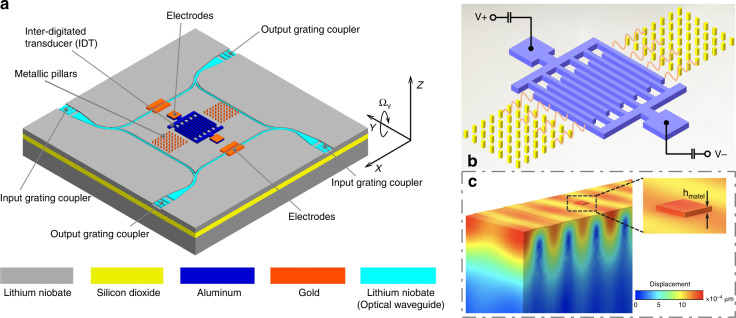
Working principle of the new progressive wave gyroscope. **a** The proposed progressive wave gyroscope consists of IDTs, metallic pillars, electrodes, and optical waveguides. The structure uses the acousto-optic effect to convert strain in the substrate material induced by SAWs to a change of effective refractive index of the waveguide, so as to optically detect the angular velocity of the gyroscope. **b** The alternating electrical voltage is applied to the vertical metallic electrodes to excite the SAW, and this SAW is transmitted along the metallic pillars on both sides. **c** The metallic pillars located at the antinode of the SAW can ensure the codirectional enhancement of the Coriolis force.

As demonstrated in Fig. 1a, the new progressive wave gyroscope based on the acousto-optic effect mainly consists of IDTs, metallic pillars, sputtered electrodes, and optical waveguides etched on the surface of the substrate thin film. The progressive wave mode generated by the IDTs is propagated along the X axis. When the out-of-plane rotation Ω along the *Y* axis is applied on the device, the metallic pillars located on the propagation path generate the secondary SAW due to the effect of the Coriolis force, and its propagation direction is the same as that of the progressive wave. The mechanical strain induced by SAWs change the refractive index of the optical waveguide.

The optical signal is injected into the waveguide with the grating coupler, and the phase of the optical signal is transmitted in the optical waveguide variation under the acousto-optic effect, causing the output optical signal to change in intensity. We can monitor the light intensity variation to detect the angular velocity of the gyroscope. To better reveal the formation principle of progressive waves, Fig. 1b depicts a schematic of single-electrode bidirectional IDTs. These structures consist of a sequence of vertical metallic electrodes or fingers connected to two bus bars, which generate alternating electric fields on the device. The key geometric parameters for SAW gyroscopes are shown in supplementary Table S1. The inverse piezoelectric effect generates tension and compression strains on the surface of the substrate, which causes each pair of fingers to excite radiated SAWs to both sides under the electric field. The wavelength of the SAW is determined by the finger period of the IDTs, and its propagation velocity depends on the properties of the substrate material and the direction of transmission. The material of the device is thin-film LN-on-insulator (LNOI) wafers to enhance the acousto-optic effect. When compared with the traditional lithium niobate, LNOI has a higher refractive index and conductivity, which has the benefit of improving the sensitivity of the SAW gyroscope. The relationship between the operating frequency and propagation velocity is expressed as follows:

1 $$f_{SAW} = \frac{{v_{SAW}}}{{\lambda _{SAW}}}$$

where *f*_*SAW*_ is the resonant frequency, *v*_*SAW*_ is the SAW velocity, and *λ*_*SAW*_ is the acoustic wavelength. When the alternating electrical voltage applied to the electrodes on both sides of the IDT is close to the resonant frequency *f*_*SAW*_, the IDT excites a stable SAW that propagates to both sides. To enhance the Coriolis force effect, the metallic pillars need to be distributed on the propagation path of the progressive wave and be located on the antinode of the SAW, as shown in Fig. 1c. This arrangement enables the pillars to vibrate in the same way, ensuring the enhancement effect of the Coriolis force in the same direction.

**Fig. 2:**
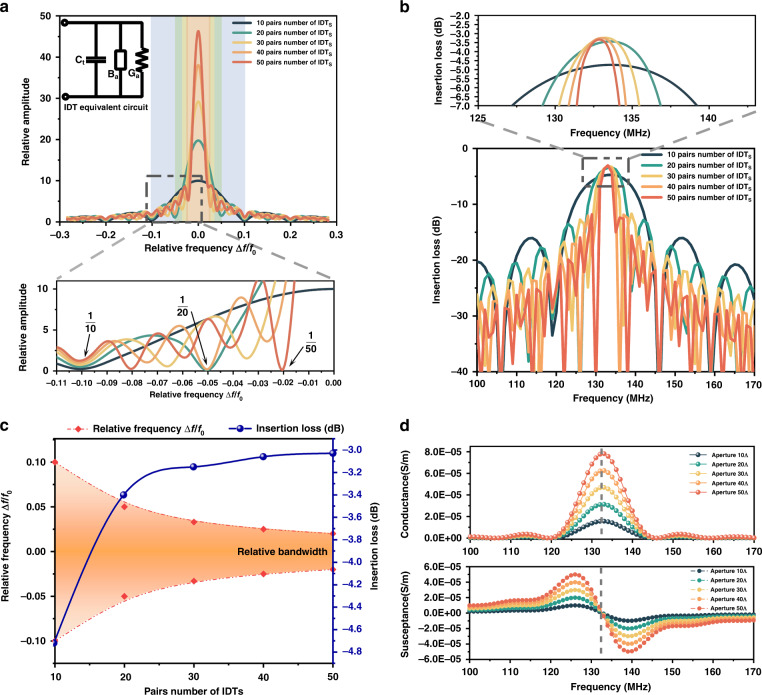
IDT characteristics. Using IDT equivalent circuits to analyze their characteristics. **a** IDT frequency characteristics with different pair numbers. **b** IDT insertion loss characteristics. **c** The number of IDT pairs has an effect on both working bandwidth and insertion loss. The left axis shows the relative bandwidth dependency on the numbers of IDT pairs. The right axis shows the loss insertion curve with different pair numbers. **d** Influence of IDT conductance and susceptance under different acoustic apertures.

The proposed new progressive wave gyroscope can be divided into an acoustic excitation module, which generates the progressive wave mode, and an optical detection module, which measures the vibration signal from the optical waveguide. Here, we employ Rayleigh waves as the excitation mode of the gyroscope and extract the vibration characteristics of the Rayleigh wave mode through mode analysis (see supplementary section 2). The operating frequency of the SAW device can be calculated by $f_{SAW} = \left( {f_{sc - } + f_{sc + }} \right)/2$, with a design value of 132.7 MHz. As can be seen from the three-dimensional model in Fig. S1, the SAW is generated through IDTs, whose characteristics have important influences on the transmission of the wave. The illustration in Fig. 2a shows the equivalent circuit of the IDT, which mainly consists of the equivalent resistance, conductance, and inductance. Assuming that all IDT energy can be converted into acoustic radiation energy, the radiation energy on both sides of the IDT under the unit pulse excitation can be simplified as follows^38^:

2 $$E\left( f \right) = 8K^2C_sf_{SAW}N^2\left| {\frac{{\sin X}}{X}} \right|^2exp^2\left( {\frac{{ - 2\pi jfN}}{{2f_{SAW}}}} \right)$$

where *K*^2^ is the electromechanical coupling coefficient for surface waves, *C*_*s*_ is the capacitance of each pair IDT, *f*_*SAW*_ is resonant frequency, *f* is the sweep frequency, *N* is the number pairs of IDTs, *X* can be expressed as *X = Nπδf/f*_*SAW*_, and *δf* is the frequency detuning.

Based on the input admittance and radiation susceptance, the insertion loss (IL) can be calculated to characterize the SAW transmission, which is expressed as follows:

3 $$IL = - 10log\left( {\frac{{2G_a\left( f \right)R}}{{\left( {1 + G_a\left( f \right)R} \right)^2 + \left( {R\left( {2\pi fC_t + B_a\left( f \right)} \right)} \right)^2}}} \right)$$

where *R* is impedance, *G*_*a*_ *(f)* is the real part in the frequency domain, *B*_*a*_ *(f)* is the radiation susceptance, and *C*_*t*_ is the capacitance of IDTs. According to Eq. (2), we can obtain the frequency response curve under various pairs of IDTs, as shown in Fig. 2a. The amplitude–frequency curve presents a functional relationship of *sinX⁄X*. The output amplitude reaches the maximum in the state of resonance, and the greater the number of IDT pairs, the narrower the frequency response bandwidth is. As the frequency response curve of the IDTs is axially symmetric, only the response of the left part is analyzed, as shown in the enlarged figure in Fig. 2a. When the number of IDT pairs is set to ten, the intersection of the frequency characteristic curve and X axis (that is, the frequency of zero point) is $\Delta f/\left( {10 \cdot f_{SAW}} \right)$. When the number changes to twenty, the frequency of zero point is $\Delta f/\left( {20 \cdot f_{SAW}} \right)$. Due to the symmetry of the frequency response curve, there are symmetrical zero points on the -X axis. The range between the two zero points is defined as the relative frequency response bandwidth. It can be found that the relative frequency response bandwidth is inversely proportional to the number of IDT pairs, which can explain the fact that increasing the number of pairs leads to narrower frequency response bandwidth. Figure 2b illustrates the insertion loss calculated according to Eq. (3). When the number of IDT pairs is 50, the maximum insertion loss at the resonant frequency of 132.7 MHz is −3.02 dB. The number of IDT pairs affect not only the range of the frequency response bandwidth but also the intensity of insertion loss. Therefore, it is necessary to make a tradeoff between the frequency response bandwidth and the insertion loss during structure design. Figure 2c shows the relationship between the number of pairs, frequency response bandwidth, and insertion loss. The results indicate the decrease of insertion loss with an increase in the number of IDT pairs, at the cost of narrower working bandwidth. When the number of pairs is set below 20, the insertion loss increases significantly, resulting in overall device performance degradation. If the number is over 40, it leads to a narrower working bandwidth, making the gyroscope work beyond the bandwidth. As a compromise between lower loss insertion and relatively high working bandwidth, the number of IDT pairs is 40 in the present design.

Figure 2d shows the analysis of IDT conductance and susceptance with various acoustic apertures. Conductance represents the efficiency at which SAWs convert electrical energy into mechanical energy. With the increase of the acoustic aperture, the peak of conductance at the resonant frequency gradually rises, while the susceptance remains zero, which indicates that the IDTs completely convert electrical energy into mechanical energy without storing it, achieving the best conversion efficiency. Therefore, the driving frequency of the IDTs should be close to the resonant frequency, and the acoustic aperture should be set as much as possible when designing the structure.

### The sensitivity of the SAW gyroscope

SAWs generated by IDTs are transferred to the optical waveguide after passing through the metal pillars, leading to changes in the refractive index of the medium. When the external rotational angular velocity Ω exists and the device rotates along the *Y* axis, the metal pillars generate Coriolis force *F*_*c*_ along the X axis, which can be expressed as follows:

4 $$F_c = - 2M_p{{\Omega }} \times v_p = - 2M_p{{\Omega }} \times \delta \sqrt {\frac{{P_mQ_D}}{{\pi f_{{{{\mathrm{SAW}}}}}M_r}}}$$

where *M*_*p*_ is the total mass of the metallic pillars, *P*_*m*_ is the excitation power, *Q*_*D*_ is the quality factor of the SAW excitation part, *M*_*r*_ is the equivalent mass of the resonator, and δ is the coefficient of transverse wave relative to longitudinal wave.

The mechanical sensitivity of the acoustic process for the SAW gyroscope *SF*_*m*_ can be obtained as follows (see supplementary section 3):

5 $$SF_m = \frac{{\partial \left( {\Delta n_c} \right)}}{{\partial {{\Omega }}}} = \frac{1}{2}\left( {n_0 + \sqrt {\frac{{M_2 \cdot 10^7P_a}}{{2lH}}} } \right)^3p_{eff}\frac{{ - 2M_p}}{{\rho v_{SAW}^2lH}} \cdot \delta \sqrt {\frac{{P_mQ_D}}{{\pi f_{{{{\mathrm{SAW}}}}}M_r}}}$$

where ∆*n*_*c*_ is refractive index variation induced by the Coriolis force, *n*_0_ is the initial refractive index of the waveguide material, *M*_2_ is the material’s acousto-optic figure of merit, *P*_*a*_ is the acoustic power, *ρ* is the substrate mass density, *l* is the length of the acoustic aperture, *H* is the SAW penetration depth, and *p*_*eff*_ is the effective acousto-optic coefficient in the specific propagation direction of the SAW.

The strain caused by the SAW can be compressive or tensile, leading to an increase or decrease in local refractive index, respectively, since the strain changes both the number of microscopic dipoles per unit volume and the microscopic potential. Specifically, the variation of refractive index Δ*n*_*i*_ (*i* *=*  *x, y, z*) in three orthogonal directions can be given by^39^:

6 $$\left[ {\begin{array}{*{20}{c}} {\Delta n_x} \\ {\Delta n_y} \\ {\Delta n_z} \end{array}} \right] = - \frac{1}{2}n_0^3\left[ {\begin{array}{*{20}{c}} {P_{11}} & {P_{12}} & {P_{13}} \\ {P_{12}} & {P_{11}} & {P_{13}} \\ {P_{31}} & {P_{31}} & {P_{33}} \end{array}} \right] \cdot \left[ {\begin{array}{*{20}{c}} {S_x} \\ {S_y} \\ {S_z} \end{array}} \right]$$

where ∆*n*_*i*_ refers to the variation in the refractive index for light polarized linearly along the *i* direction, *n*_0_ is the original refractive index of the material without any strain, and *S*_*i*_ is the strain applied in the *i* direction.

**Fig. 3:**
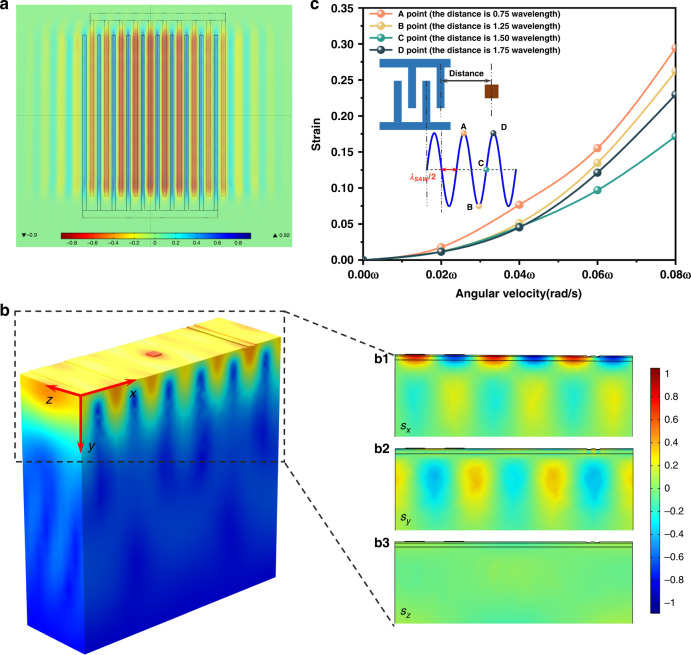
Strain distribution induced by SAWs. **a** The SAW is generated by applying an alternating electrical signal to the metal electrode on the interdigital transducer and transmits it to both sides. (The cross fingers in the red part of the picture indicate that the applied voltage is positive, and the cross fingers in the blue part of the picture indicate that the applied voltage is negative.) **b** Schematic diagram of strain distribution (**b**_**1**_ is strain distribution in the x direction, S_X_; **b**_**2**_ is strain distribution in the y direction, S_Y_; **b**_**3**_ is strain distribution in the z direction, S_Z_). **c** SAWs at different positions result in different strains.

By applying an alternating electrical signal to the metal electrode on the IDT, an electric field is generated on the surface of the substrate covered by the IDT. As shown in Fig. 3a, under the inverse piezoelectric effect, SAWs are excited on the substrate and transmit to both sides, leading to mechanical strain on the substrate surface; the cross fingers in red indicate the compressive strain, and the cross fingers in blue indicate the tensile strain. Figure 3b shows the strain distribution, which is generated by the fundamental SAW when a voltage signal with an amplitude of 5 V is applied to the IDT’s electrode. The top, middle, and bottom panels in Fig. 3b represent the induced strain in the x, y, and z directions, respectively. Since Rayleigh SAWs are polarized waves, their particles move in a plane perpendicular to the direction of propagation, and the trajectory is elliptical, which leads to greater tensile and compression strains in the transmission process of SAWs in the x and y directions. This is consistent with the simulation results in the top panel of Fig. 3b, where the tensile and compressive strains are mainly concentrated on the surface of the material, which is clearly different from the strain energy distribution in the middle panel. The middle panel shows that the strain distribution of the surface is mostly concentrated in a single SAW wavelength. Furthermore, the strain distribution generated in the z direction shown in the bottom panel is almost zero. This result indicates that the influence of the induced strain in the x direction *S*_*x*_ and the y direction *S*_*y*_ on the refractive index is significantly higher than that in the z direction, which provides a reference for the subsequent installation of the SAW gyroscope.

The strain distribution induced by SAWs also depends on the location of the metal pillars and the waveguide. In the illustration in Fig. 3c, we select four sampling points at different locations, whose distances from the IDTs are 0.75*λ*_*saw*_(peak), 1.25*λ*_*saw*_(trough), 1.5*λ*_*saw*_(node), and 1.75*λ*_*saw*_(peak), respectively. By applying the external rotational angular velocity to the device, the strain at different points can be obtained. According to the results, Point A, at the peak, has the largest strain variation under different rotation angular velocities, followed by Point B, at the trough, and Point C, at the node. In addition, the strain variation at Point D is smaller than that at Point A. This is because the energy of the SAW dissipates in the process of transmission, leading to a smaller strain. Therefore, to maximize the strain on the waveguide, it should be placed as close as possible to the peak or trough of the SAW.

**Fig. 4:**
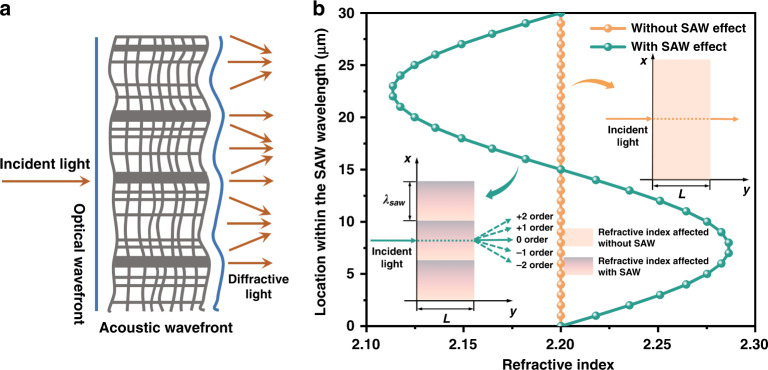
Raman–Nath diffraction characteristics. **a** Schematic diagram of Raman–Nath diffraction. **b** Refractive index variation of the medium within a period of *λ*_*SAW*_ with and without SAW affect. The modulated refractive index variation is expressed as follows: $\Delta N_i = \left| {{{\Delta }}n_i} \right|\sin \left( {K_{SAW}x - \omega _{SAW}t} \right)$, where $K_{SAW} = 2\pi /{\uplambda}_{SAW}$ and $\omega _{SAW} = 2\pi f_{SAW}$.

The strain induced by SAWs, which are a kind of mechanical wave, in the transmission process causes the medium to alternate with density, like a phase grating. The gyroscope proposed in this paper is based on the principle of the acousto-optic effect, using the intensity of diffracted light, which is changed by the SAW field to detect external rotational angular velocity. According to the Klein–Cook parameter Q^40^, the structural parameters determine the diffraction state as Raman–Nath diffraction (see supplementary section 4). The designed gyroscope resonant frequency is 132.7 MHz, and the acoustic aperture of the IDTs is same as the acousto-optic interaction length. Therefore, the acoustic aperture of the IDTs is 900 μm in the present structure. As shown in Fig. 4a, with the influence of SAWs, the lattice of the medium be stretched or compressed, resulting in a decrease or increase in density, changing the refractive index of the medium and forming a refractive index modulation grating with the period of *λ*_*SAW*_. Since the SAW’s velocity is much smaller than that of light, the refractive index modulated grating can be considered static. When the light passes through the medium in parallel, it is subject to phase modulation, that is, the optical wavefront passing through the dense part of the medium is delayed, while the optical wavefront passing through the loose part of the medium is advanced. Therefore, the optical wavefront in the area affected by the SAW appears concave and convex shapes and becomes a rhythmic “wrinkle” surface (blue line). The secondary waves emitted by the wavelet source on the surface of the outgoing wave are mutually coherent, resulting in multilevel diffraction light symmetrically distributed with respect to the incident light, that is, Raman–Nath diffraction. Figure 4b shows the refractive index variation of the medium within a period of *λ*_*SAW*_. In the absence of SAWs, the refractive index remains constant (orange line), while in the presence of SAWs, the refractive index of the medium has a sinusoidal relationship with the location of the SAW wavelength (green line).

The expression between the optical signal intensity and refractive index of the gyroscope under the influence of SAW is as follows (see supplementary section 3.2):

7 $$SF_t = \frac{{\partial \left( {I_2 - I_1} \right)}}{{\partial \left( {\Delta n_c} \right)}} = \varepsilon _0cE_1^2$$

where *I*_*2*_ and *I*_*1*_ are the light intensity affected by the SAW with and without Ω, respectively, *E*_1_ is the input light field intensity, *ε*_0_ is the permittivity of vacuum, and *c* is the speed of light in free space.

By detecting the changes in light intensity, the information of the external rotational angular velocity can be quantified, and the angular velocity signal of the gyroscope can be measured. Therefore, the mechanical sensitivity of the acousto-optic gyroscope can be expressed as follows:

8 $$SF = SF_t \cdot SF_m = \varepsilon _0c\left( {n{^{\prime}}} \right)^3P_{eff}\frac{{ - E_1^2M_pv_p}}{{\rho v_{SAW}^2lH}}$$

**Fig. 5:**
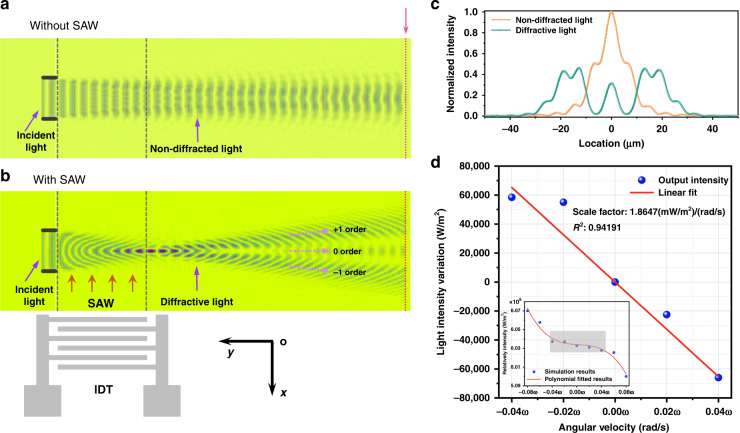
Simulation of acousto-optic interaction. Optical electric field profiles from COMSOL simulation **a** without the SAW and **b** with the SAW. **c** Characteristics of light intensity transmitted in optical waveguides under different conditions. **d** Rotational angular velocity—light intensity output curve of the acousto-optic gyroscope.

For our proposed progressive wave acousto-optic gyroscope, the angular velocity signal is detected via optical waveguide. When the SAW is applied to the optical waveguide, the mechanical strain changes the refractive index of the waveguide, which affects the output optical power intensity. Due to wafer defects and crystal orientation, the refractive index of sensing waveguide and reference waveguide are not exactly the same after fabrication. To reduce measurement error, electro-optical effect is used to fine tune the refractive index of the reference waveguide. Figure 5a, b show the comparison of simulation results without and with SAWs, respectively. For our device, the light is coupled into the chip by a fiber and guided through a rib optical waveguide that is defined by two etched grooves on the LN thin film. The optical light is injected into the rib waveguide from the left side, and the input light beam then propagates as a two-dimensional beam in the LN thin film. Compared with the free-running state of the waveguide, the presence of the SAW changes the refractive index of waveguide and then causes scattering of the light, which weakens the optical power intensity of the output port (right side). Figure 5c shows that the power intensity of the waveguide output with an SAW accounts for approximately 35% compared to that without an SAW, and that the reduced power intensity is transmitted to the diffraction light order. In addition, as the external rotational angular velocity increases, the refractive index change caused by the SAW gradually increases, which further attenuates the optical intensity. The external rotational angular velocity can be obtained by detecting the optical intensity. The illustration in Fig. 5d shows the relationship between the output light intensity of the acousto-optic gyroscope and its rotational angular velocity. The simulated sensitivity of the acousto-optic gyroscope is 1.8647 (mW/m^2^)/(rad/s) within a range of ±0.04ω. By normalization, the sensitivity of the acousto-optic gyroscope can be enhanced by four orders of magnitude compared to that of the traditional SAW gyroscope. Simultaneously, according to the analysis of chip damage under overload, the proposed acousto-optic gyroscope can withstand a 220,000 g impact (see supplementary section 5), which provides a feasible and insightful solution for performance enhancement of angular velocity measurement.

## Discussion

In this paper, we propose a new strategy to improve the sensitivity of sensor by using the acousto-optic effect instead of traditional SAW gyroscopes. However, many factors affecting the performance of sensor should be considered in practical applications. First, the output signal of traditional SAW gyroscopes is very weak, usually at the millivolt level, which severely affects the practical use and performance of sensors. Thus, in the subsequent experimental tests, we need to build the weak signal measurement circuit, and use electronic components such as an amplifier, filter and lock-in amplifier to extract the weak signal of the sensor. Especially in optical detection, the noise and temperature control of photoelectric detector are also important factors affecting the final performance of the sensor. In addition, the coupling efficiency between optical fiber and waveguide directly affect the sensor performance. The cross-section area difference between optical fiber and waveguide is too large, which leads to a large amount of energy loss in optical measurement^41^. In general, the core diameter of a single-mode fiber is approximately 8~10.5 μm^42^, while the cross-section area of a single-mode waveguide is approximately 1.0~0.64 μm^2^ ^43^. Therefore, we need to use grating coupling calibration device to reduce the insertion loss caused by the misalignment between the fiber and waveguide, and then improve the coupling efficiency. Another important factor affecting the performance is temperature, which affects the propagation speed of the SAW and thermal expansion of the piezoelectric crystal and ultimately causes the resonant frequency of the SAW resonator to drift^44^. Thus, the piezoelectric crystal with low velocity-temperature coefficient and thermal expansion coefficient can be selected as the material of the SAW resonator to reduce the sensitivity of the resonant frequency to temperature. Moreover, hardware and software compensation can be applied to minimize the influence of temperature on the performance of the SAW sensor, such as algorithmic compensation^45^ and on-chip micro-oven temperature control^46^.

## Conclusion

In this paper, a new progressive wave gyroscope based on acousto-optic effects is demonstrated. The mechanical strain generated by SAWs is transformed into light intensity for detection, utilizing the acousto-optic effect in the design process. The simulation results show that the sensitivity of the structure is 1.8647 (mW/m^2^)/(rad/s) and the impact load resistance can reach 220,000 g. Compared with the traditional SAW gyroscope based on frequency detection, the sensitivity of the structure can be improved by four orders of magnitude; this shows its superiority. A comparison of main specifications between our device and reported ones is given in Table 1. The gyroscope in this work has a relatively higher sensitivity, small size, and greater impact resistance. According to the theoretical and finite element simulation results, the mechanical sensitivity of the proposed structure has a certain dependence on its geometric parameters, so the sensitivity of the gyroscope can be further improved by optimizing the geometric parameters of the structure. The proposed acousto-optic gyroscope is suitable for angular velocity signal detection in extreme environments such as aerospace, military strikes, and geological exploration and could play an important role in rocket launching and oil drilling.

**Table 1:** Comparison of performances of some of the reported SAW gyroscopes with our designed devices.

Reference	Die area (mm^2^)	Frequency (MHz)	Scale factor	Normalized scale factor	Shock
^17,18^	1.2 × 0.75	80	119 Hz/deg/s	1.4875 × 10^−6^/deg/s	/
^16^	9 × 9	98.6	0.431 Hz/deg/s	0.437 × 10^−8^/deg/s	/
^47^	1.4 × 0.6	80.2	52.35 Hz/deg/s	0.6527 × 10^−6^/deg/s	/
^48^	/	30	43 Hz/deg/s	1.4333 × 10^−6^/deg/s	/
^7^	7.8 × 7.8	115.17	0.959 µV/deg/s	1.19875 × 10^−6^/deg/s	50,000 g
^49^	20 × 20	115.5	0.025 µV/deg/s	0.5 × 10^−7^/deg/s	50,000 g
This work	8 × 8	132.7	1.8647 (mW/m^2^)/(rad/s)	0.4013 × 10^−2^/deg/s	220,000 g

## Supplementary information

Supplemental information
