# Supplementary material for: A novel progressive wave gyroscope based on acousto-optic effects
Source: Microsyst Nanoeng. 2022 Sep 2;8:95. doi: 10.1038/s41378-022-00429-4 (PMC9437048; doi:10.1038/s41378-022-00429-4)
Supplement: Supplementary file 1 — Supplemental information [file 41378_2022_429_MOESM1_ESM.docx]

**Supplementary Information for “A Novel Progressive Wave Gyroscope Based on Acousto-optic Effects”**

**1. The Geometric Parameters of the Surface Acoustic Wave Gyroscope**

For the surface acoustic wave gyroscope based on acousto-optic effect, the geometric parameters have crucial influence on the whole sensor performance, the value of the key parameters are listed in the table S1.

**Supplementary** **Table S1.** The key geometric parameter for the surface acoustic wave gyroscope

| **Parameters** | **Value** |
| --- | --- |
| IDTs finger period | 30μm |
| IDT pairs number | 40 |
| Thickness of IDTs | 200nm |
| Metallic pillars area | 7.5μm×7.5μm |
| Thickness of Metallic pillars | 500nm |
| Metallic pillars number | 200 |
| Distance between IDTs and metallic pillars | 112.5μm/127.5μm |
| Metallic pillars interval | 30μm |
| Waveguide width | 2μm |
| The thickness of thin film LN | 500nm |

**2. The schematic diagram of acoustic excitation module**

In order to understand the vibrating modes of devices driven by SAWs, a finite element model of the structure is built by the commercial software COMSOL Multiphysic. Fig. S1a shows the simulation model of the acoustic excitation module, which is used to analyze the transmission characteristics and vibration modes of the SAW on the substrate. Considering the anisotropy of LN thin film on insulator SiO_2_, LNOI with high refractive index difference is used in this work, and its properties are transformed by Euler Angle with the aim of obtaining more precise simulation results. Since the IDT is the periodic structure, the periodic boundary condition is adopted to simplify the model for reducing computation, and the propagation characteristics of SAWs in LNOI are studied in a single period. The Rayleigh SAW has high transduction efficiency because its energy is mainly concentrated in a single SAW wavelength. Here, we employ Rayleigh waves as the excitation mode of the gyroscope and extract the vibration characteristics of the Rayleigh wave mode through mode analysis, as shown in Figs. S1b and S1c. Due to the reflection effect of the IDT with periodic arrangement, the eigenfrequency of the SAW is divided into two components to generate the stopband; of these, one is the lower-boundary frequency of Stopband A with a frequency of *f_sc-_*, and the other is the upper-boundary frequency of Stopband B with a frequency of *f_sc+_*; thus, the operating frequency of the SAW device can be calculated by $f_{SAW}={(f_{sc-}+f_{sc+})}/2$. In this paper, the operating frequency of SAW is 132.7 MHz.


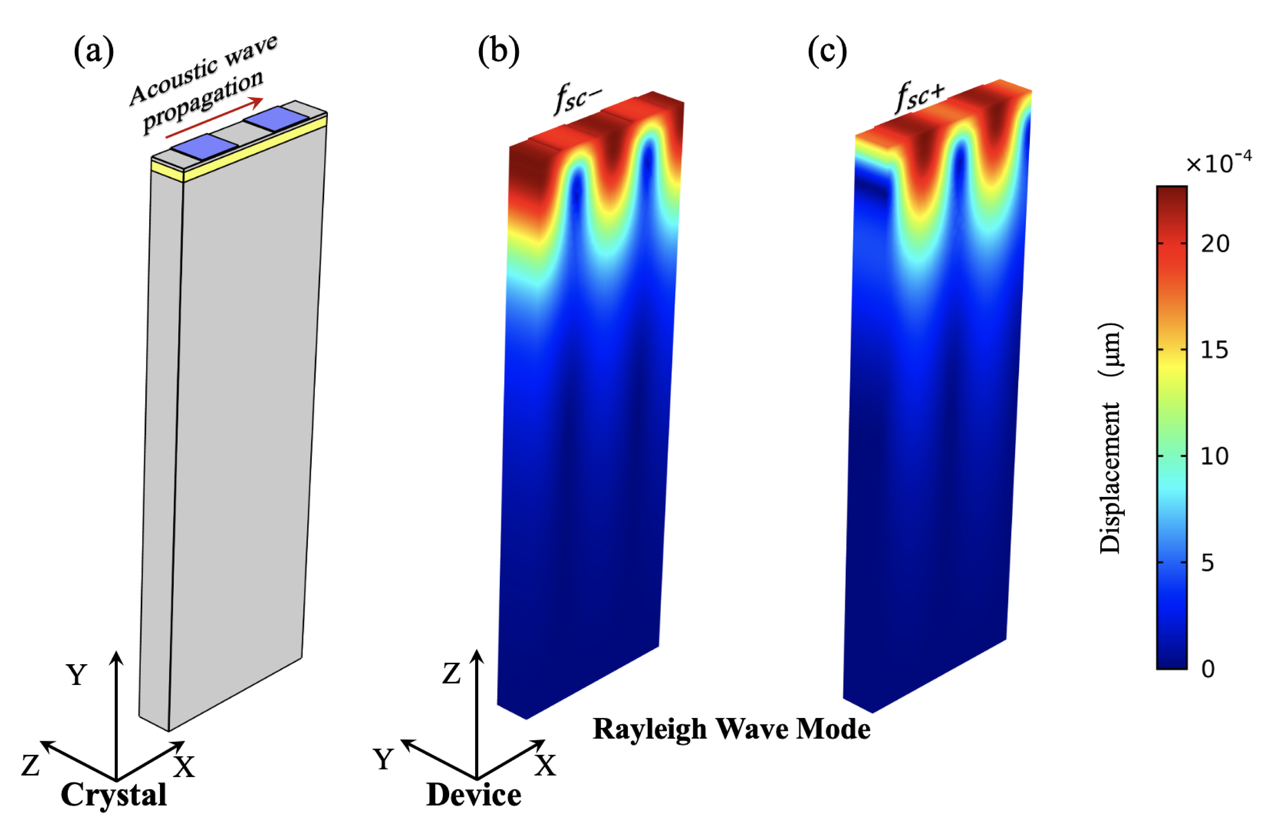


**Fig. S1. 3D simulation schematic diagram of acoustic excitation module.** (a) IDTs are used to excite SAWs on LNOI with high refractive index difference, and the excitation mode is generated through the progressive wave. (b) and (c) represent two stopband boundary frequencies. The vibration characteristic of Rayleigh wave modes can be extracted through mode analysis, and the eigenfrequency is divided into a lower-boundary frequency *f_sc-_* and an upper-boundary frequency *f_sc+_* of the stopbands due to the reflection of periodic IDTs.

**3. The theoretically sensitivity of the SAW gyroscope**

**3.1 The mechanical sensitivity of acoustic process**

Surface acoustic waves generated by IDTs will be transferred to the optical waveguide after passing through the metal pillars, which will lead to changes in the refractive index of the medium:

$n^{'}=n_{0}+\Delta n$ (S1)

where $n_{0}$ is the initial refractive index of the waveguide material, $\Delta n$ is the variation of the refractive index without external rotational angular velocity, and $\Delta n$ can be expressed as follows:

$\Delta n=\sqrt{\frac{M_{2}{10}^{7}P_{a}}{2lH}}$ (S2)

where $M_{2}$ is the material’s acousto-optic figure of merit, $P_{a}$ is the acoustic power, *l* is the length of the acoustic aperture, and *H* is the SAW penetration depth.

When the external rotational angular velocity Ω exists and the device rotates along the Y axis, the metal pillars will generate Coriolis force along the X axis, which can be expressed as follows:

$F_{c}=-2M_{p}\Omega\times v_{p}=-2M_{p}\Omega\times\delta\sqrt{\frac{P_{m}Q_{D}}{\pi f_{\mathrm{SAW}}M_{r}}}$ (S3)

where $M_{p}$is the total mass of the metallic pillars, $P_{m}$ is the excitation power, $Q_{D}$ is the quality factor of the SAW excitation part, $M_{r}$ is the equivalent mass of the resonator, and δ is the coefficient of transverse wave relative to longitudinal wave.

As the strain of Rayleigh SAW mode is expressed by stress, which is directly related to Coriolis force, the relationship between strain *S* and Coriolis force is as follows:

$S=\frac{F_{c}}{\rho{v_{SAW}}^{2}lH}$ (S4)

where $\rho$ is the substrate mass density.

The refractive index variation $\Delta n_{c}$ induced by the Coriolis force can be obtained as follows:

$\Delta n_{c}=\frac{1}{2}\left( n^{'} \right)^{3}p_{eff}\frac{-2M_{p}\Omega}{\rho{v_{SAW}}^{2}lH}\cdot\delta\sqrt{\frac{P_{m}Q_{D}}{\pi f_{\mathrm{SAW}}M_{r}}}$ (S5)

where $p_{eff}$ is the effective acousto-optic coefficient in the specific propagation direction of the SAW.

Due to the existence of Coriolis force, the refractive index will change again under its effect; this is called the second variation of refractive index $n^{'}'$.

$n^{''}=n^{'}+\Delta n_{c}$ (S6)

Therefore, the mechanical sensitivity of acoustic process for the SAW gyroscope ${SF}_{m}$ can be obtained as follows:

${SF}_{m}=\frac{\partial\left( \Delta n_{c} \right)}{\partial\Omega}=\frac{1}{2}\left( n_{0}+\sqrt{\frac{M_{2}{10}^{7}P_{a}}{2lH}} \right)^{3}p_{eff}\frac{-2M_{p}}{\rho{v_{SAW}}^{2}lH}\cdot\delta\sqrt{\frac{P_{m}Q_{D}}{\pi f_{\mathrm{SAW}}M_{r}}}$ (S7)

**3.2 The mechanical sensitivity of photonic process**

In this work, we propose a differential waveguide structure to enhance the sensitivity of gyroscope. Fig. S2a shows the optical waveguide topology design scheme with only a pair of couplers, which includes one input coupler (Input) and two output couplers (Output1/Output2). When the optical signal is injected from the input port, the Y-connection structure divides it into two and transmits it independently from the two optical waveguides to the respective output port. Transverse optical waveguide (sensing waveguide) is subjected to SAW and Coriolis force, and the refractive index will be changed. On the contrary, the refractive index of the longitudinal optical waveguide (reference waveguide) remains constant without SAWs, which can be used as a reference signal. Obviously, the angular velocity can be measured by comparing the light intensity of the two output couplers.


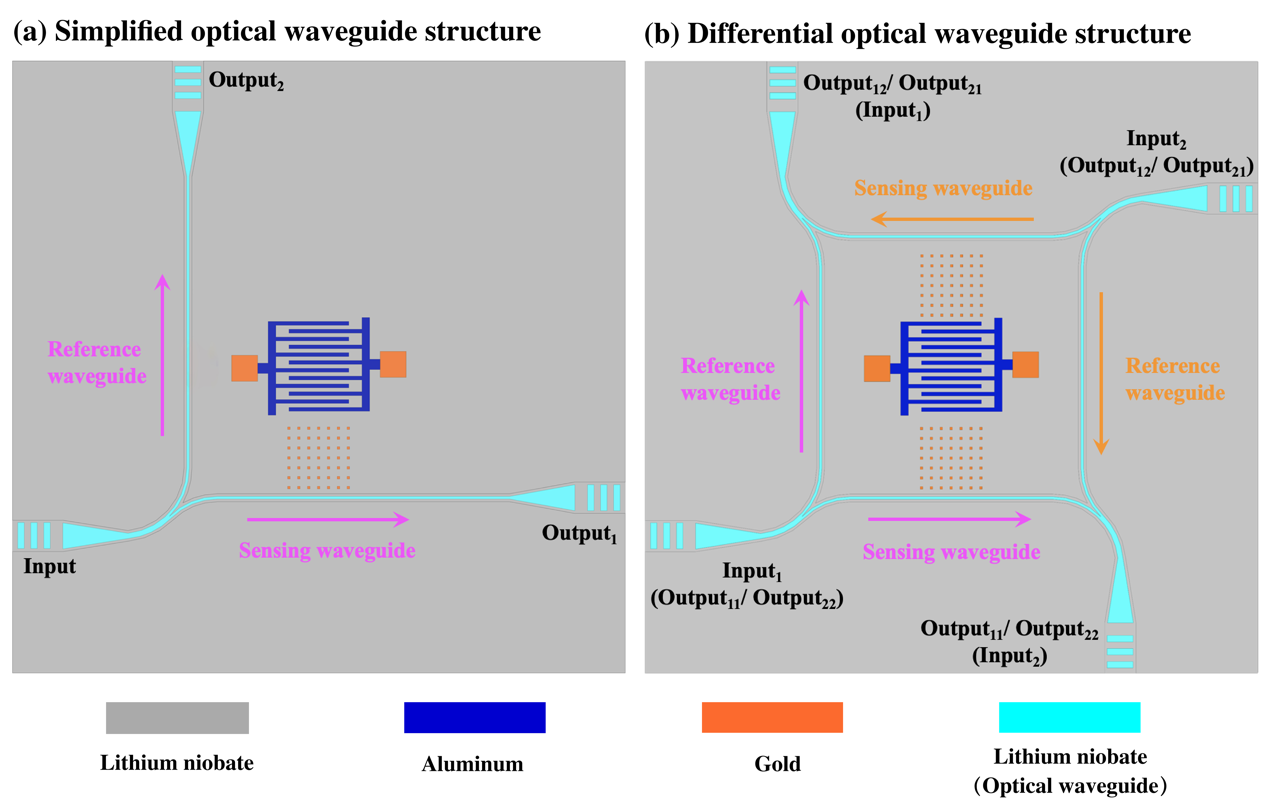


**Fig. S2. Comparison of different optical waveguide configuration schemes.** (a) Simplified optical waveguide structure. (b) Differential optical waveguide structure.

In order to further improve the sensitivity of the acousto-optic gyroscope, we designed a differential optical waveguide structure on the basis of Fig. S2a, and the structure of the waveguide is shown in Fig. S2b. The differential structure contains two input ports and two output ports. The metallic pillars are located on either side of the IDT to provide opposite Coriolis forces. In order to ensure the same strength of Coriolis force between adjacent metallic arrays, the spacing should be an integer times of half wavelength. In the direction of SAW propagation, the interval between adjacent pillar is one wavelength, and the co-vibration of pillars ensure the co-directional enhancement of Coriolis force. In order to ensure that the metallic pillars on the both sides of IDT has opposite Coriolis force, the distance between metallic pillars and the edge of IDT differs by half wavelength. As shown in Fig. S3, the distance of metallic pillars on both sides to the left edge and right edge of IDT is ${3\lambda_{SAW}}/4$ and ${\lambda_{SAW}}/4$ respectively. The opposite Coriolis force on the both sides of metallic pillars will lead to the opposite strain change, which will cause the opposite refractive index variation of optical waveguide. By comparing and differentiating with the respective reference signals, the sensitivity of the sensor can be enhanced by up to 2 times.


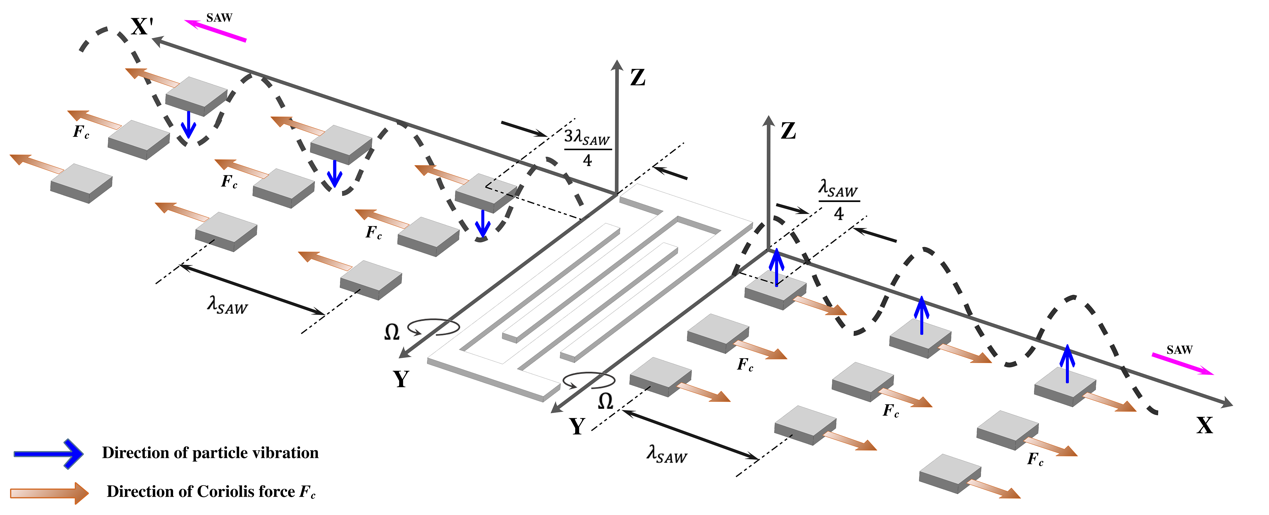


**Fig. S3. Metallic pillars configuration scheme for differential structure.**

The theoretical sensitivity of differential waveguide structures will be derived below. In Fig. S2b, the two sensing waveguides will be subjected to Coriolis forces in opposite directions, which will cause the refractive index of sensing waveguide to change in opposite trends. Thus, the refractive index variation $\Delta n_{c}$ induced by the Coriolis force can be obtained as follows:

$\Delta n_{c}=\frac{1}{2}{(n^{'})}^{3}P_{eff}\frac{F_{c}}{\rho v_{SAW}^{2}lH}$ (S8)

Due to the existence of external rotational angular velocity, the secondary variation of refractive index $n^{''}$ of two sensing waveguides under Coriolis force can be expressed by,

$n_{1}^{''}=n_{0}+\Delta n+\Delta n_{c}$ (S9)

$n_{2}^{''}=n_{0}+\Delta n-\Delta n_{c}$ (S10)

In the acoustic-optic gyroscope, we utilize the light intensity to detect the refractive index variation. When the Coriolis force is applied to the optical waveguide, the mechanical strain will change the refractive index of the waveguides, which affects the output light intensity. The time averaged intensity of light, can be related to the magnitude of the electric field, by the relationship

$I=\frac{1}{2}\varepsilon_{0}cnE^{2}$ (S11)

where $E^{2}$ is the magnitude of electric field, $\varepsilon_{0}$ is the permittivity of vacuum, and $c$ is the speed of light in vacuum, $n$ is the refractive index of optical waveguide.

The difference between the output light intensity of sensing waveguide and reference waveguide can be expressed as

$I_{2}-I_{1}=\frac{1}{2}\varepsilon_{0}cn^{''}E_{2}^{2}-\frac{1}{2}\varepsilon_{0}cn^{'}E_{1}^{2}$ (S12)

Where $I_{1}$, $I_{2}$ are the output light intensity of reference and sensing waveguide, $E_{1}^{2}$, $E_{2}^{2}$ are the electric field of reference and sensing waveguide respectively.

Considering that the secondary variation of refractive index is very small, we assume that the electric files of reference waveguide and sensing waveguide are approximately equal ($E_{1}^{2}\approx E_{2}^{2}$). Substitute Eq. (S8) into Eq. (S12) and simplify, the relationship between light intensity and Coriolis force is obtained by,

$I_{2}-I_{1}=\frac{1}{4}\varepsilon_{0}c{(n')}^{3}P_{eff}E_{1}^{2}\frac{F_{c}}{\rho v_{SAW}^{2}lH}$ (S13)

Considering the differential waveguide structure, the total mechanical sensitivity of acoustic-optical gyroscope can be expressed as follow,

$SF=\frac{\partial\left( I_{2}-I_{1} \right)}{\partial\left( \Omega\right)}=\varepsilon_{0}c{(n')}^{3}P_{eff}\frac{-E_{1}^{2}M_{p}v_{p}}{\rho v_{SAW}^{2}lH}$ (S14)

where $M_{p}$is the total mass of the metallic pillars, $P_{m}$ is the excitation power,

The advantages of designing two pairs of couplers are that the differential structure can be used to improve the performance of sensors. Not only that, but this approach also significantly enhances the utilization of I/O couplers. In the subsequent experimental tests, the input and output ports can be flexibly exchanged, which will greatly improve the interchangeability of the structure.

**4. The Klein-Cook Parameter**

The gyroscope proposed in this paper is based on the principle of acousto-optic effect, using the intensity of diffracted light, which is changed by the SAW field to detect external rotational angular velocity. The acousto-optic effect is divided into two distinct diffraction states: Raman–Nath and Bragg diffraction. The Klein–Cook parameter Q is used to distinguish the states:

$Q=\frac{2\pi\lambda L}{n_{0}\lambda_{SAW}^{2}}$ (S15)

where $\lambda$ and $\lambda_{SAW}$ are the wavelength of incident light in vacuum and SAWs in the medium, respectively, and *L* is the acousto-optic interaction length. Theoretically, when $Q\ll1$, Raman–Nath diffraction occurs; otherwise, Bragg diffraction occurs instead (shown in the red part of the Fig. S4b). However, being limited to the ratio of optical wavelength and SAW wavelength ($\lambda/{\lambda_{SAW}}$), we generally utilize $Q=4\pi$ to distinguish the diffraction states.

According to Eq. (S15), Fig. S4a demonstrates the interactive relationship between the acousto-optic interaction length *L*, $\lambda/{\lambda_{SAW}}$, and *Q* value. When $\lambda/{\lambda_{SAW}}$ increases from 0 to 0.3 and *L* varies from 2000 to 0, the *Q* value monotonously increases. While designing the device structure, we usually reduced the interaction length *L* as much as possible to enhance the refractive index variation, but this leads to the ratio of optical wavelength and SAW wavelength $\lambda/{\lambda_{SAW}}$ having to be increased, which will pose a huge challenge for IDT manufacturing. In our designed structure, the structural parameters determine the diffraction state as Raman–Nath diffraction (${2\pi\lambda L}/{n_{0}\lambda_{SAW}^{2}}$=4.43<$4\pi$). The designed gyroscope resonant frequency is 132.7 MHz, and the acoustic aperture of the IDTs is same as the acousto-optic interaction length. Therefore, the acoustic aperture of the IDTs is 900μm in the present structure.

**
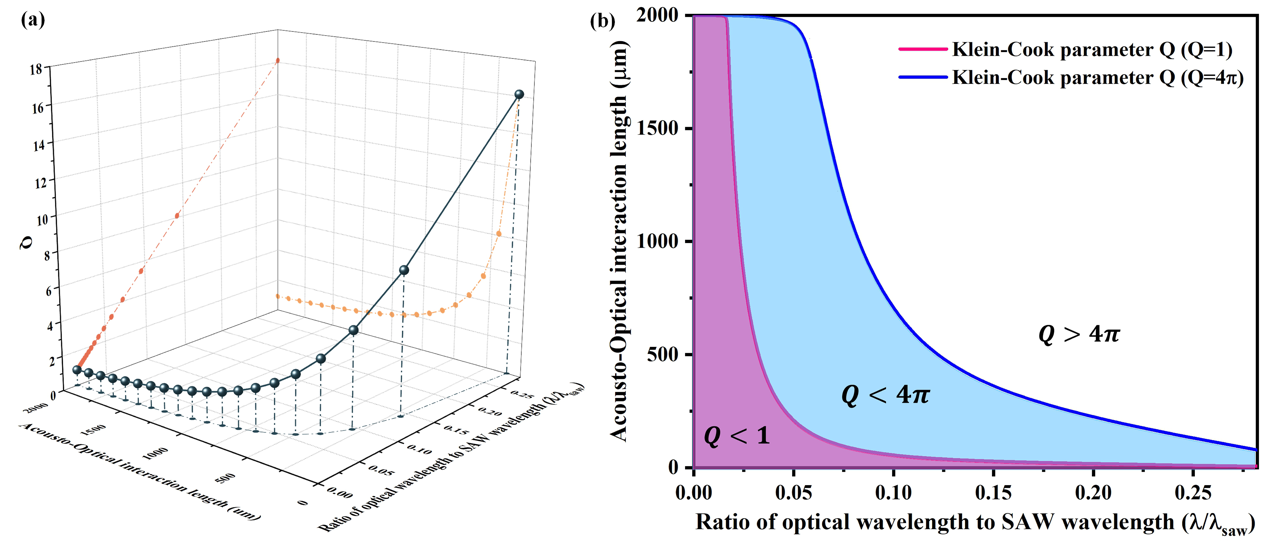
**

**Fig. S4. Determination of acousto-optic diffraction type.** (a) The parameter Q is affected by the length of acousto-optic interaction, the SAW wavelength and the optical wavelength. (b) Theoretically, Raman–Nath diffraction occurs when Q < 1; however, Raman–Nath diffraction conditions can be satisfied when Q < 4$\pi$ in practical application.

**5. Shock**

The shock effect is an important factor to be considered in the design process of gyroscopes intended for applications in harsh environments. In order to analyze chip damage under overload, COMSOL simulation is carried out to calculate the stress distribution of the device; the results are shown in Fig. S5. As can be seen from Fig. S5a, the maximum stress is found near the four corners of the substrate, while the stress everywhere else on the device is not as high. This is because the acoustic sensitive part and the optical detection part are formed by depositing or etching on LiNbO_3_. They have no suspended movable parts, which improves the impact resistance of the device.

Fig. S5b shows that the maximum stress of the device increases as the impact load increases. Since the tensile fracture range of LiNbO_3_ is between 8MPa and 37.5MPa, it can be said that when the maximum stress is less than 8MPa (shown in the green part of the Fig. S5b), the chip will not be damaged, but when it is subjected to an impact load of more than 220,000g, the maximum stress of the device exceeds the tensile fracture range of LiNbO_3_, and the edge of the sensor will be broken. Therefore, it is reasonable to conclude that the proposed acousto-optic gyroscope can withstand a 220,000g impact.


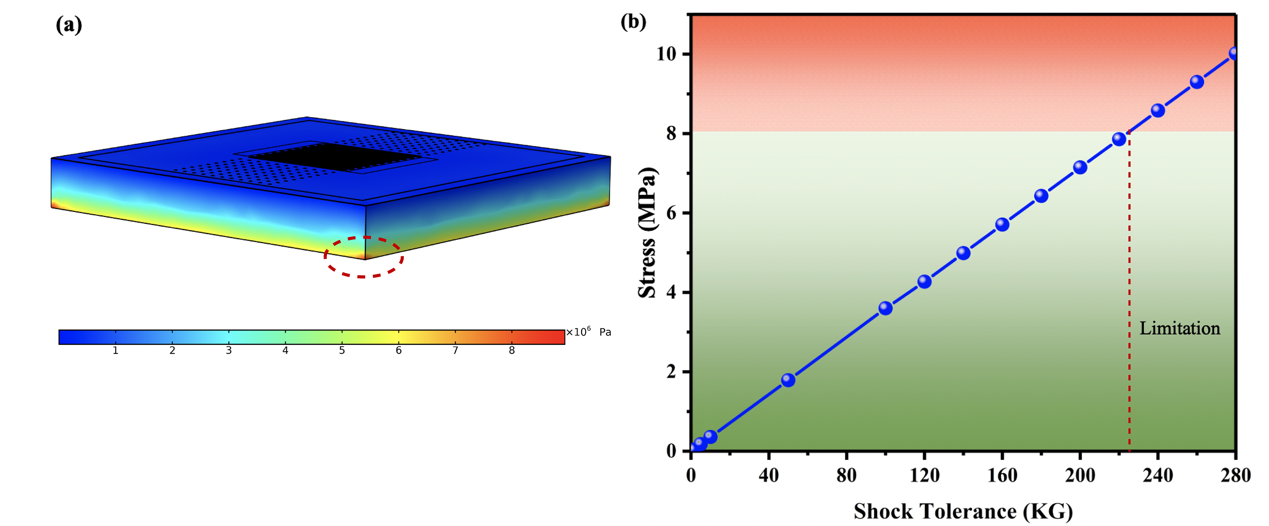


**Fig. S5. The impact load on the device.** (a) The maximum stress on the device will occur near the four corners of the substrate. (b) With the increase of impact load, the maximum stress of the device gradually increases. When the maximum stress exceeds 8MPa, the sensor is damaged.
